# Population Genetic Structure of the Grasshopper *Eyprepocnemis plorans* in the South and East of the Iberian Peninsula

**DOI:** 10.1371/journal.pone.0059041

**Published:** 2013-03-08

**Authors:** María Inmaculada Manrique-Poyato, María Dolores López-León, Ricardo Gómez, Francisco Perfectti, Juan Pedro Martínez Camacho

**Affiliations:** 1 Departamento de Genética,Universidad de Granada, Granada, Spain; 2 Departamento de Células Troncales, Centro Andaluz de Biología Molecular y Medicina Regenerativa (CABIMER), Sevilla, Spain; 3 Departamento de Ciencia y Tecnología Agroforestal, Universidad de Castilla La Mancha, Albacete, Spain; University of Lausanne, Switzerland

## Abstract

The grasshopper *Eyprepocnemis plorans* subsp. *plorans* harbors a very widespread polymorphism for supernumerary (B) chromosomes which appear to have arisen recently. These chromosomes behave as genomic parasites because they are harmful for the individuals carrying them and show meiotic drive in the initial stages of population invasion. The rapid increase in B chromosome frequency at intrapopulation level is thus granted by meiotic drive, but its spread among populations most likely depends on interpopulation gene flow. We analyze here the population genetic structure in 10 natural populations from two regions (in the south and east) of the Iberian Peninsula. The southern populations were coastal whereas the eastern ones were inland populations located at 260–655 m altitude. The analysis of 97 ISSR markers revealed significant genetic differentiation among populations (average G_ST_ =  0.129), and the Structure software and AMOVA indicated a significant genetic differentiation between southern and eastern populations. There was also significant isolation by distance (IBD) between populations. Remarkably, these results were roughly similar to those found when only the markers showing low or no dropout were included, suggesting that allelic dropout had negligible effects on population genetic analysis. We conclude that high gene flow helped this parasitic B chromosome to spread through most of the geographical range of the subspecies *E. plorans plorans*.

## Introduction

Supernumerary chromosomes, also called accessory or B chromosomes, are additional chromosomes found in some individuals at some populations of about 15% of eukaryotic species [1]. Most of them are harmful for the host genome and invade populations at the expense of a variety of drive mechanisms [2].The grasshopper *Eyprepocnemis plorans* subsp. *plorans* lives in moist habitats near river courses and coastal areas throughout the circum-Mediterranean region, also reaching the Caucasus and the Arabian Peninsula [3]. B chromosomes have been found in almost all natural populations of this subspecies so far analyzed from Spain, Morocco, Italy, Greece, Turkey, Armenia, and Dagestan [4], the only B-lacking region being located at the headwaters of the Segura River basin in Spain [5]. A recent study has suggested that B chromosomes in *E. plorans plorans* are rather young [6]. It would thus be helpful to understand how these B chromosomes have spread so rapidly throughout most of the geographical range of this subspecies. The existence of meiotic drive for B chromosomes in this species [7] explains how they rapidly invade new populations [8]. However, ascertaining how B chromosomes reached other populations would require the knowledge of population genetic structure, since this would provide indirect estimations of the magnitude of gene flow among populations.

Gene flow may be directly analyzed by demographic studies, e.g. capture-mark-recapture methods, but it can also be indirectly estimated through population genetic analysis by molecular markers. In this latter case, the calculation of Wright's indices to estimate inbreeding at several hierarchical levels (*F_IS_*, *F_ST_* and *F_IT_*) from observed intrapopulation heterozygosities as well as expected population and metapopulation heterozygosities, is the most commonly used tool [9], [10]. A number of molecular markers can be used to gain information on population genetics and structure [11], microsatellites (SSRs) being one of the most commonly used because of a number of advantages, such as their co-dominant nature and a high degree of polymorphism. However, the use of SSRs requires a prior process of ascertaining the appropriate primers to amplify them by PCR, which implies the sequencing of DNA regions that are adjacent to each SSR. In organisms with little or no DNA-sequence information, an easier alternative is to use theoretical SSR sequences to design PCR primers that are able to amplify the DNA regions between two consecutive SSRs. The size (in base pairs) of these amplicons constitutes the Inter Simple Sequence Repeat (ISSR) markers [12]. These behave as dominant, with "band presence" corresponding to the dominant phenotype (homozygous or heterozygous) and "band absence" being the recessive phenotype. Although this is a clear disadvantage because it impedes a direct estimation of some population parameters (e.g. observed heterozygosity and thus Wright's F_IS_ and F_IT_), they have certain advantages: the ease of generating a high number of markers, because no previous genomic information is necessary; relatively high reproducibility; and the possibility of visualizing them on agarose gels [13].

ISSR markers have been used for a variety of genetic and population-genetic purposes, such as: the characterization of different varieties in maize [14], wheat [13], and barley [15]; the genetic characterization of several organisms [16]; the analysis of genetic diversity [17], [18]; the identification of genes associated with disease resistance [19]; phylogenetic analysis [20], [21]; and gene mapping [22]. In insects, ISSR markers have been employed to characterize different lineages in *Bombyx mori* [23] and *Antheraea mylitta* [24], as well as in studies on hybridization [25], genetic diversity [26]–[28], and conservation genetics [29].

One of the main caveats of ISSR markers, and other markers based on PCR amplification, is allelic dropout, i.e. a stochastic failure of PCR amplification in one of the alleles carried by an individual at a given locus, leading to the determination of a wrong genotype [30]. Even though ISSR markers show Mendelian inheritance [31], allelic dropout can yield an excess of the "band absence" allele which could be mistaken for segregation distortion. Therefore, it is advisable to evaluate the effect of allelic dropout on the PCR repeatability and inheritance of ISSR markers [32], [33].

In this paper, we analyze several parameters of population genetics and structure in 10 natural populations of the grasshopper *Eyprepocnemis plorans* subsp. *plorans* by means of ISSR markers, and also evaluate the incidence of allelic dropout on these parameters. The populations were sampled at two Spanish geographical regions, one being coastal and located in the south and the other being inland and located in the east, including several populations from the B-lacking region in the Segura River basin. The B chromosome variant found in the eastern region (B_1_) is different from those found in the southern region (B_2_ and B_24_), but all of them share the same kinds of repetitive DNAs [34]. The only population-genetic analysis performed previously in this species used B-chromosome frequency along several rivers in eastern Spain, including the Segura, Júcar, and Turia rivers [35]. These authors provided the first evidence for isolation by distance (IBD) in this species, suggesting that B-chromosome dynamics can best be explained by metapopulation models. Our present results confirm the existence of IBD in this species and show a clear genetic differentiation among populations belonging to the two regions analyzed. In addition, the low Gst values observed help explain how this young B-chromosome system has attained such a widespread geographical distribution.

## Results

### Genetic variation and population structure

The six primers employed yielded a total of 97 ISSR markers ranging in size from 180 to >2000 bp in the 10 populations as a whole, with 99% of the loci being polymorphic. Per region, however, the percentage of polymorphic loci fell to 77.9% (SD =  3.13) in the southern populations and 76.7% (SD =  1.78) in the eastern ones (Table 1).

**Table 1:** Proportion (SD) of polymorphic loci in the 10 populations analyzed.

		Polymorphic loci	
Region	Population	Number	%
South	Algarrobo	76	78.4
	Torrox	79	81.4
	Nerja-0	78	80.4
	Nerja-2	73	75.3
	Salobreña	72	74.2
	Average	75.6 (3.05)	77.9 (3.13)
East	Mundo	76	78
	Claras	76	78.4
	Socovos	74	76
	Calasparra	72	74
	Caravaca	74	76
	Average (SD)	74.4 (1.67)	76.5 (1.78)

Data analysis with Hickory v1.1 [36], a program providing estimates of population genetic parameters through a Bayesian approach, pointed to both *full* and *f = *0 as the best models, as indicated by the DIC parameters [37] (Table S1). However, the *full* model indicated an inbreeding value (0.96) being highly unlikely for a polygynandric organism like *E. plorans* [38]. For this reason, we chose the *f = 0* (no inbreeding) for all subsequent calculations.

Table 2 shows that θ^(II)^, an analogous of Nei's G_ST_, was 0.129, i.e. very similar to the Bayesian estimate of this latter parameter (Gst-B = 0.123), and 95% confidence intervals indicated that both were significantly higher than zero. These low G_ST_ values indicate that genetic diversification of these populations by drift is impeded by high gene flow. An AMOVA showed the existence of significant population structure, with 9.61% variation among populations within regions (φ_SC_) (P<0.0001), 15.05% between the southern and eastern regions (φ_CT_) (P<0.001) and the remaining 75.34% found within populations (φ_ST_) (Table S2).

**Table 2:** Population parameters estimated by Hickory under the *f = 0* model.

Parameter	Mean	SD	2.50%	97.50%
theta-I, θ^(I)^	0.299	0.021	0.26	0.344
theta-II, θ^(II)^	0.129	0.008	0.114	0.145
theta-III, θ^(III)^	0.098	0.004	0.091	0.105
hs[Algarrobo]	0.229	0.005	0.219	0.239
hs[Torrox]	0.23	0.005	0.219	0.24
hs[Nerja-0]	0.218	0.005	0.208	0.228
hs[Nerja-2]	0.228	0.005	0.219	0.238
hs[Salobreña]	0.229	0.005	0.218	0.24
hs[Mundo]	0.235	0.007	0.221	0.249
hs[Claras]	0.22	0.007	0.207	0.233
hs[Socovos]	0.205	0.006	0.193	0.216
hs[Calasparra]	0.21	0.005	0.199	0.22
hs[Caravaca]	0.216	0.007	0.203	0.23
Hs	0.222	0.002	0.218	0.226
Ht	0.253	0.002	0.249	0.257
Gst-B	0.123	0.005	0.114	0.133

2.5% and 97.5% show confidence interval

Note that hs values are estimates of genetic diversity (panmictic or expected heterozygosity) for each population sample, whereas Hs is the average of hs values for all population samples. Ht is the heterozygosity that would be observed if all population samples would come from a single population.

**Figure 1:**
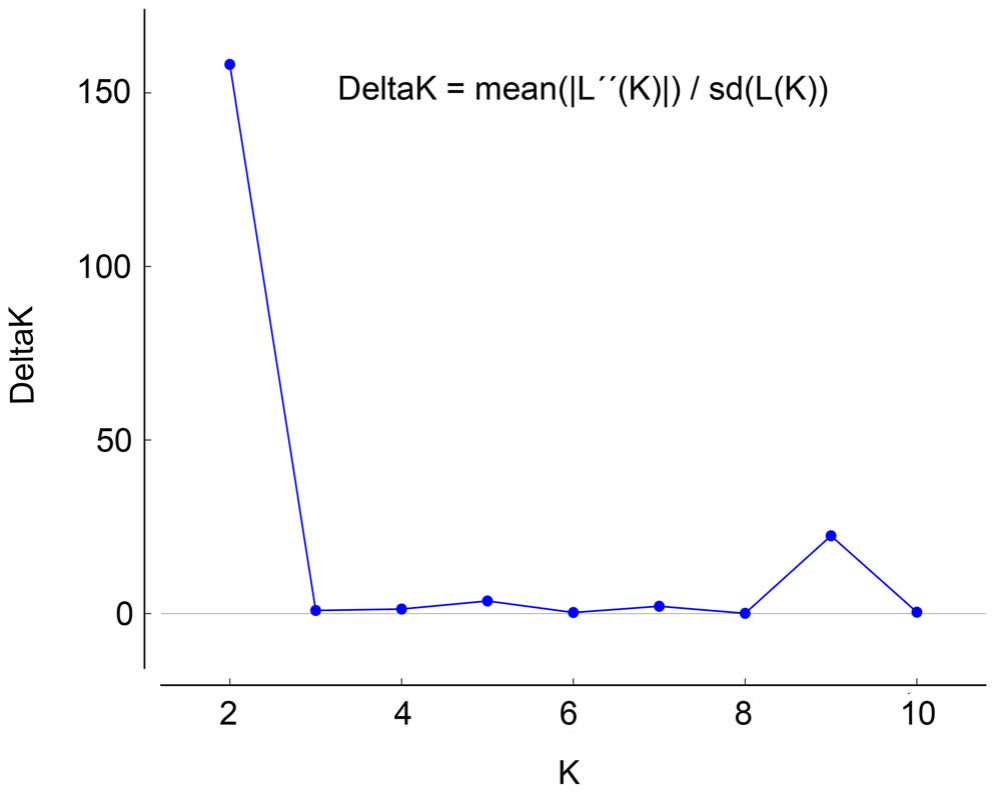
Delta K values with respect to K, according to the calculation method by Evanno et al. [42]. These results were found using all 97 ISSR markers analyzed. Note the highest peak for K =  2.

**Figure 2:**
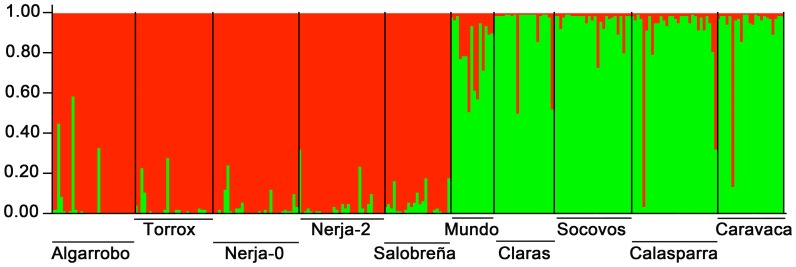
Ancestry of each individual to any of the two groups, using all 97 ISSR markers analyzed, yielded by the Structure software. Each vertical bar represents one of the 255 individuals analyzed. Group 1 (southern region) is represented in red color, and Group 2 (eastern region) is shown in green color. Bar length is proportional to the inferred ancestry values into each group for each individual.

The Structure software, which analyzes genetic structure through a Bayesian approach, showed that the number of genetic groups (K value) best fitting our data, inferred following Evanno *et al*. [39], was K = 2 (Figure 1 and Table S3), with most individuals from the southern populations being included in the Group 1 and most individuals from eastern populations being in the Group 2 (Table 3). This indicates that southern and eastern populations constitute two genetically differentiated groups (Figure 2).

**Table 3:** Proportion of individuals from each population assigned to each group by the Structure software, with K =  2.

Population	N	Group 1	Group 2
Algarrobo	29	0.940	0.060
Torrox	27	0.964	0.036
Nerja-0	30	0.968	0.032
Nerja-2	30	0.962	0.038
Salobreña	23	0.949	0.051
Mundo	15	0.170	0.830
Claras	21	0.058	0.942
Socovos	27	0.037	0.963
Calasparra	30	0.091	0.909
Caravaca	23	0.064	0.936

The highest assignment proportion to each group is marked in bold.

The Mantel test revealed significant isolation by distance (IBD) when we used pairwise-Fst values (r =  0.90, P =  0.0004) or Dice's dissimilarity indices (r =  0.68, P =  0.00003) for genetic distance (see genetic and geographical distances in Tables S4 and S5).

**Figure 3:**
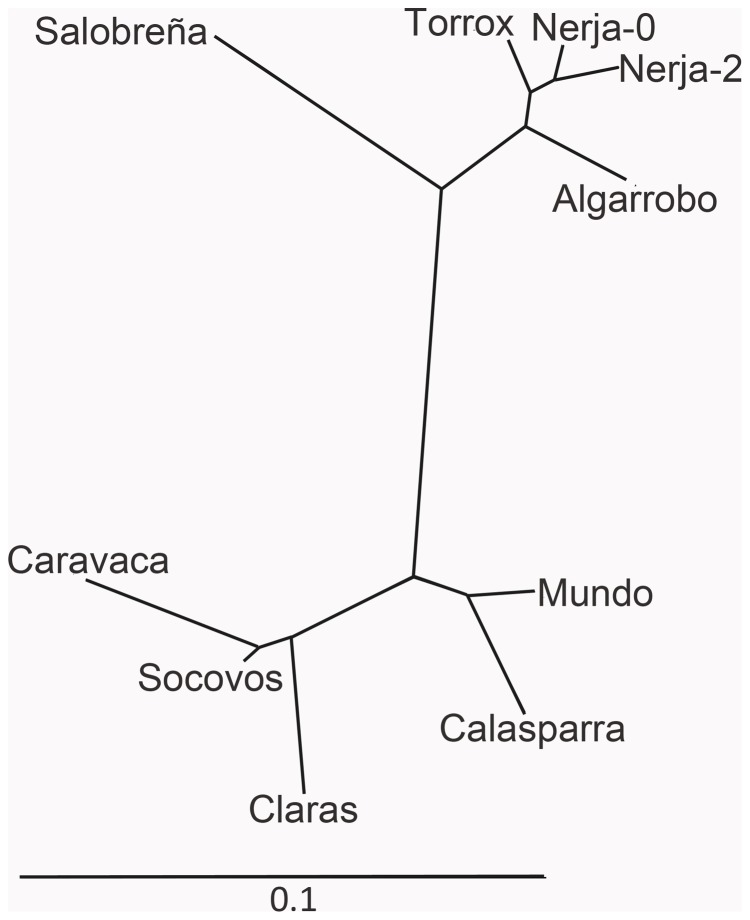
Unrooted Neighbor-Joining phylogenetic tree. This was built with Pairwise-Fst genetic distances determined with the 97 ISSR markers analyzed. Note the two groups of populations corresponding to the southern (upper half) and eastern (lower half) regions. All populations harbored B chromosomes excepting Caravaca, Socovos and Claras.

An unrooted neighbor-joining phylogenetic tree built with the *Pairwise-Fst* genetic distances (drawn with the AFLP-surv software), showed the longest distance between the southern and eastern regions (Figure 3).

### Repeatability and inheritance of ISSR markers

More than half of the ISSR markers (51 out of 97) displayed consistent patterns under the two controls performed, but 3 markers failed to show repeatability under control 1 only, 40 failed for control 2 only, and 4 markers failed for both controls (Table S6). Only six markers (26-500, 26-650, 26-750, 26-1000, 39-250, and 43-540) showed a frequency of allelic dropout higher than 30%. Control 2 revealed significantly higher levels of dropout than control 1 (t =  5.8, df =  97, P<0.0001). Correlation analyses showed no significant association between fragment size and dropout measured in controls 1 (r =  -0.05, N =  98, P =  0.65) or 2 (r =  -0.15, N =  98, P =  0.13).

A total of 101 progeny analyses (PAs) were performed to test inheritance of 23 ISSR markers (Table S7). As a whole, we found significant departures from Mendelian proportions in 30/90 PAs. To summarize these results, we applied a heterogeneity chi-square test for markers where more than one PA had been performed for a same type of cross. This decreased the number of PAs to 49, 20 of which showed P<0.05. However, only five of these remained significant after the sequential Bonferroni test (three 1x1 and two 0x1 crosses). Remarkably, these five PAs showed an excess of marker-lacking individuals, as expected from allelic dropout. However, among the 15 PAs that lost significance with the sequential Bonferroni test, 8 showed an excess of marker-lacking embryos but the remaining 7 showed an excess of marker-carrying embryos, these figures being consistent with chance effects. It bears noting that four of the five ISSR markers whose inheritance was tested in the five PAs rejecting the null hypothesis on segregation (i.e. 7-1000, 7-1100, 7-1400 and 14-750) had manifested allelic dropout in the PCR test (see Table S6), whereas it had not been observed for 14-1450. In addition, 7 out of the 10 markers involved in the PAs that lost significance after correction for multiple tests, had shown a dropout in the PCR tests. We therefore consider that our results on the inheritance of the 23 ISSR markers analyzed failed to show significant departures from Mendelian segregation that were not caused by allelic dropout.

### Population parameter estimates after discarding ISSR markers showing allelic dropout

To analyze the possible incidence of allelic dropout on population genetics and structure results, we repeated calculations with Hickory and Structure after discarding the 10 markers showing high incidence of dropout detected by the PCR repeatability analysis (26-500, 26-650, 26-750, 26-1000, 39-250 and 43-540) or inheritance analysis (7-1000, 7-1100, 7-1400 and 14-750). The population parameters estimated with the 87 remaining markers, listed in Table S8, gave results almost identical to those found with all 97 markers (compare with Table 2). Even using only the 46 markers showing no sign of allelic dropout (after discarding those that failed PCR repeatability or showed segregation distortion), population genetic estimates differed by only 8.16–29.7% from those made with all markers (Table S9).

The analysis of population structure with the 87 ISSR markers showing slight dropout or none at all, with the Structure software, gave very similar results to those found when including all the markers, with K =  2 best fitting our data, and high proportions of individuals from the southern populations being included in one group and those from the eastern populations in the other group (see Table S10 and Figures S1 and S2).

Even with only 46 markers, Structure showed that K =  2 best fitted our data, and the genetic differentiation between the southern and eastern regions was clearly evident, with high proportions of individual assignment to the correct region (see Table S11 and Figures S3 and S4).

## Discussion

As in other organisms previously analyzed [23], [25], [28], [40], ISSR markers are highly polymorphic in the grasshopper *E. plorans*, with 99% of the loci showing variation in the 10 populations as a whole, and more than 75% of loci being variable in each population analyzed. Since ISSR markers are dominant, only expected heterozygosity (hs) can be calculated (assuming Hardy-Weinberg equilibrium), and it ranged between 0.205 in Socovos and 0.235 in Mundo. These values are similar to those described for ISSR markers in other insects such as, for instance, *Antheraea mylitta* [24] and *Plutella xylostella* [28]. Much lower values were found for ISSR markers in the hymenopteran *Gonatocerus ashmeadi* [27], but higher values have been reported for RAPDs in the South American grasshopper *Sinipta dalmani* (0.274–0.326) [41] and the mosquito *Anopheles darlingi* (0.27–0.32) [42].

Hs (i.e. the average for hs in all populations) was lower than Ht (expected heterozygosity in all populations as a whole), suggesting a possible heterozygous deficiency which could be explained by allelic dropout, inbreeding or population subdivision. As explained above, dropout effects are negligible in this case, and inbreeding is unlikely since *E. plorans* is a polygynandric species [38] showing large population size. Unfortunately, Wright's Fis, which would provide an estimate of inbreeding, cannot be calculated with dominant markers. With this concern in mind, we conclude that, in this case, heterozygous deficiency is most likely explained by population subdivision, given that the 10 population samples included two geographically well-separated subsets.

The significant values found for θ^(II)^ and Gst-B, and the highest variance between regions, compared to within regions, revealed the existence of population subdivision for the analyzed areas. Population structure was also shown by the Structure software. A K of 2 best explained the data, with population groups being well defined in coincidence with the southern and eastern regions, so that more than 90% of individuals were properly assigned to them. Remarkably, our analysis of population genetics and structure parameters, after discarding those markers showing allelic dropout, showed almost identical results to those performed with the complete data set, suggesting that allelic dropout has a minor effect on these estimations. Therefore, although ISSR, and other markers being affected by allelic dropout, become less relevant with the advent of new sequencing technologies and genotyping methods, they still constitute an efficient and low-cost method of revealing population structure, especially in non-model organisms.

In the 10 populations of the grasshopper *E. plorans* analyzed here, the low (but significant) values of G_ST_ found suggest that gene flow is high in these populations, but not high enough to avoid a certain genetic differentiation between southern and eastern populations. The grasshopper *E. plorans* is more a jumper than a flyer, but it has a high dispersal ability facilitated by its polyphagy with low dietary demand [43]. The slight prevalence of gene flow over drift is thus logical, bearing also in mind the significant IBD found among the populations analyzed.

The only previous analysis of F_ST_ and IBD in a grasshopper species, performed in the South American grasshopper *Sinipta dalmani* (using RAPD markers), revealed extensive variation for Fst (0.058–0.23, mean =  0.122), i.e. slightly higher than those detected by us for the ISSR markers in *E. plorans*, but no significant IBD was found among the 8 populations analyzed in *S. dalmani* [41]. These authors explained this lack of IBD by non-gradual divergence among the populations analyzed. Crickets in the genus *Stenopelmatus* are other orthopterans in which significant IBD has been revealed with ISSR and mtDNA markers [44]. The significant IBD observed in *E. plorans* for the ISSR markers is consistent with previous IBD shown, in this same species, for B-chromosome frequency [35].

The high gene flow inferred from the low G_ST_ values observed for these ten populations of *E. plorans plorans* from the south and east of the Iberian Peninsula, contributes to explain the rapid spread of parasitic B chromosomes throughout most of its geographical range. B chromosomes are genomic parasites of strictly vertical transmission [45]. Therefore, although they can prosper in a given population, reaching high frequency at the expense of meiotic drive [8], gene flow is necessary to augment their spread across populations. Our present analysis, showing high levels of gene flow among populations of the grasshopper *E. plorans plorans*, in accord with the high dispersal ability reported for this species [43], explains why these B chromosomes have reached almost the entire geographical range of this subspecies, despite being rather young [6].

Whereas the five populations from the southern region (Algarrobo, Torrox, Nerja-0, Nerja-2 and Salobreña) all carried B chromosomes, only two (Mundo and Calasparra) from the eastern region carried them. In this latter region, the observed pairwise Fst values (see Table S4) indicate almost double amount of average genetic distance between populations differing in B chromosome presence (0.09) than that found between populations non differing at this respect (0.049). For instance, Calasparra (+B) and Caravaca (-B) are the two closest populations in the eastern region (20 Km) but show one of the highest pairwise Fst values (0.1365). This suggests higher isolation (i.e. lower gene flow) between the +B and -B populations and is consistent with the possibility that B chromosomes are absent in these latter populations because B chromosomes have not yet arrived to them [5].

## Materials and Methods

### Genetic variation and population structure

Specimens of the grasshopper *Eyprepocnemis plorans* were collected at 10 natural populations in the south and east of the Iberian Peninsula (Table 4). No specific permits were required for the field studies. The locations sampled were not privately owned or protected in any way, and this field study did not involve endangered or protected species. Individuals were frozen by immersion in liquid nitrogen and stored at -80°C until used. DNA was extracted with the kit GenElute® Mammaliam Genomic DNA minipreps (SIGMA), following the manufacturer's recommendations. DNA quantification was performed in a TBS-380 minifluorometer (Turner Biosystems) using Picogreen dye (Quant-iT™ PicoGreen® dsDNA Kit; Molecular Probes, Invitrogen).

**Table 4:** Number of individuals analyzed with different primers for ISSRs.

				Altitude	ISSR6		ISSR7		ISSR14		ISSR26		ISSR39		ISSR43	
Population	Province	Region	UTM (X–Y)	(m)	Nm	Nf	Nm	Nf	Nm	Nf	Nm	Nf	Nm	Nf	Nm	Nf
Algarrobo	Malaga	South	406506.77 – 4067270.76	4	28	–	29	–	29	–	28	–	26	–	28	–
Torrox	Malaga	South	414886.32 – 4066101.15	37	26	–	23	–	27	–	25	–	18	–	25	–
Nerja-0	Malaga	South	419750.87 – 4066921.35	3	30	–	29	–	29	–	28	–	26	–	28	–
Nerja-2	Malaga	South	423550.39 – 4068087.00	79	29	–	28	–	29	–	29	–	26	–	30	–
Salobreña	Granada	South	447096.84 – 4066013.37	1	23	–	21	–	23	–	23	–	21	–	22	–
Mundo	Albacete	East	605562.49 – 4258313.37	450	15	–	15	–	15	–	15	–	10	–	15	–
Claras	Albacete	East	568336.33 – 4242425.03	640	20	–	20	–	20	–	20	–	20	–	7	–
Socovos	Albacete	East	590227.22 – 4242649.56	655	22	5	21	4	20	5	21	5	21	4	9	3
Calasparra	Murcia	East	614230.74 – 4234235.38	260	28	–	29	–	28	–	27	–	27	–	30	–
Caravaca	Murcia	East	602055.17 – 4218476.58	570	13	10	12	10	12	10	13	9	11	9	–	–
Total					234	15	227	14	232	15	229	14	206	14	194	3

Nf = Number of females; Nm = Number of males.

ISSR markers were amplified by polymerase chain reaction (PCR) using the primers shown in Table S12. PCR was performed in a 25-µl reaction containing 1x PCR buffer, 240 &M dNTPs, 2 µM of primer, 1 U of Taq polymerase (New England, Biolabs), and 10 ng of DNA. Reaction conditions included an initial denaturing at 94°C for 3 min followed by 40 cycles at 94°C (40 s), 57°C (45 s), and 72°C (1.5 min), and a final extension at 72°C for 5 min. All PCR experiments were conducted in an Eppendorf Mastercycler ep Gradient, and PCR products were visualized in 1.5% agarose gel with SYBR Safe (Invitrogen) following the manufacturer's recommendations, and analyzed in a gel-documentation system (Gel Doc XR and ChemiDoc XRS, Biorad). Fragment sizes were determined using the HyperLadder II (Bioline) molecular-weight marker, and the presence or absence of each fragment was coded as 1 and 0, respectively.

To test the incidence of PCR errors generating the absence of bands suggesting false recessive homozygotes (allelic dropout), we performed two types of replicates for the same individual, one where the two replicates shared the same master mix and were amplified at the same time (control 1), and another where the replicates were made from different master mixes and were amplified on different days (control 2). The frequency of allelic dropout was quantified as a percentage of individuals showing inconsistent genotypes in the two replicates.

ISSR data were analyzed by Bayesian methods, using the Hickory v1.1 software [36], bearing in mind their dominant nature. Data were analyzed under four different models: i) the *full model*, where the values of population differentiation (theta, θ, analogous to Wright's Fst parameter) and inbreeding (*f*, analogous to *F_is_*) were different from zero, ii) the *f = *0 model, which assumes no inbreeding within populations, iii) the *theta = *0 model, based on the absence of population differentiation, and iv) the *f free* model, or *free model*, where values of f were chosen randomly for the analysis of the data regardless of prior information. The choice of the model was based on the DIC parameter, analogous to AIC in Bayesian model selection [37] and on the Dbar parameter, a measure of how well the model fits the data (smaller values of both are better).

The existence of isolation by distance (IBD) was analyzed by means of a Mantel Test comparing the matrix of pairwise-Fst values with the matrix of geographical distances between the sampled populations, with the Zt-win software [46] using 100,000 random repetitions. The matrix of pairwise Fst values was established by the software AFLP-surv [47]. All geographical distances between populations were calculated as the crow flies.

Also, we investigated IBD by using Dice's dissimilarity index [48], which avoids the assumption of Hardy-Weinberg equilibrium. We first calculated the similarity and dissimilarity coefficients between every two individuals with the Famd v1.25 software [49], and then summarized this information per population. With the dissimilarity coefficients calculated between every two populations, we built a matrix which was analyzed, together with the above-mentioned matrix of geographical distances, by means of the Mantel test.

To elucidate the population structure in *E. plorans*, we analyzed molecular variance (AMOVA) with the Arlequin v3.5 software [50], with 10,000 permutations and 20% maximum absence of data. We defined independent groups (regions) for southern and eastern populations.

With the genetic matrix distance (pairwise-Fst), estimated using the program AFLP-surv from the ISSR marker data, we inferred a phylogenetic tree under the Neighbor-Joining method [51] implemented in the Neighbor program included in Phylip v3.69 package [52].

To identify genetically homogeneous groups within our samples, we used a Bayesian algorithm implemented in the Structure v2.3.1 software [53], assuming the existence of K populations, or groups, characterized by a set of allele frequencies for each locus. Individuals in the sample were assigned, probabilistically, to one group or more. We used the admixture model and the allele frequencies correlated model, without prior assumptions concerning the population. For each value of K (from 1 to 11), 11 independent runs were made and, for each run, 100,000 iterations were carried out after a burn-in period of 50,000 iterations. To detect the number of genetically homogeneous groups (K) that best fit the data, we used Structure Harvester website [54], which implements the Evanno method [39].

### Inheritance of ISSR markers

To analyze inheritance of ISSR markers, we performed 10 controlled crosses analyzing the markers derived from one or more of the ISSR6, ISSR7, and ISSR14 primers (Table S13). The remaining primers were discarded for inheritance analysis because they had shown the highest values of dropout in the PCR controls (see above). In total, we analyzed 19 parents (one male was involved in two crosses) and 1920 embryo progeny.

Inheritance expectations for autosomal markers, shown in Table S14, indicate that when the two progenitors show band presence (1×1 cross), it may happen that all progeny show the marker, in which case we can infer that at least one parent was homozygous for the presence of the marker. However, both parents maybe heterozygous for the presence of marker, in which case 75% of progeny would be expected to have the marker. On the other hand, when one parent has a marker and the other does not (1×0 and 0×1 crosses), 50% of progeny would be expected to have the marker if one parent is heterozygous, or all progeny if the 1 parent is homozygous. Only progenies showing variation for marker presence/absence are suitable for statistical testing. In each cross, the null hypothesis and the expected proportions in the offspring depend on parent phenotypes (see Table S14). Expected proportions for sex-linked markers are summarized in Table S15. Unfortunately, we did not ascertain the embryo progeny sex, but the two former Tables show that, as a whole, the expected frequency of the "band presence" phenotype from every phenotypic cross was about the same for autosomal and sex-linked genes. Goodness-of-fit and heterogeneity chi-square tests were employed to detect significant departures from Mendelian inheritance, and the sequential Bonferroni test was applied to minimize type-I errors.

## Supporting Information

Figure S1 **Delta K values with respect to K, according to the calculation method by Evanno et al.** [**42**] **.** These results were found using the 87 ISSR markers showing no (or low level of) allelic dropout.
(TIF)

Figure S2 **Ancestry of every individual in either of the two groups, using the 87 ISSR markers showing no allelic dropout (or low level thereof), yielded by the Structure software.** Each vertical bar represents one of the 255 individuals analyzed. Group 1 (southern region) is represented in red color, and Group 2 (eastern region) is shown in green color. Bar length is proportional to the inferred ancestry values into each group for each individual.
(TIF)

Figure S3 **Delta K values with respect to K, according to the calculation method by Evanno et al.** [**42**] **.** These results were found using the 46 ISSR markers showing no allelic dropout.
(TIF)

Figure S4 **Ancestry of each individual to either of the two groups, using the 46 ISSR markers showing no allelic dropout, yielded by the Structure software**. Each vertical bar represents one of the 255 individuals analyzed. Group 1 is represented in red, and group 2 in green color. Bar length is proportional to the inferred ancestry values into each group for each individual.
(TIF)

Table S1 **Statistical parameters for the different models after Bayesian inference.**
(DOC)

Table S2 **Analysis of Molecular Variance (AMOVA).**
(DOC)

Table S3 **Likelihood values obtained for calculation of the number of groups (K) best fitting the data through the Evanno method. Reps =  replicates.**
(DOC)

Table S4 **Pairwise-Fst genetic distances (bottom) and geographical distances in Km (top).**
(DOC)

Table S5 **Dice's dissimilarity coefficients between populations (bottom) and standard errors (top).**
(DOC)

Table S6 **Failure rate of PCR amplification for every marker (%) under the two controls performed.** Dropout values above 30% are marked in bold. N1 and N2 indicate the number of individuals where a given allele was tested for PCR repeatability under controls 1 and 2, respectively.
(DOC)

Table S7 **Inheritance analysis of ISSR markers by means of the goodness-of-fit chi square test. 0 =  Absence of the allele, 1 =  Presence of the allele, Obs.1 =  Observed frequency of 1, exp.1 =  Expected frequency of 1. Progeny analyses showing significance after sequential Bonferroni are indicated by an asterisk.**
(DOC)

Table S8 **Population genetic parameters found with Hickory (** ***f*** **= 0 model) with 87 loci, i.e. after removing the 10 ISSR markers showing the highest dropout values.** Note that hs values are estimates of genetic diversity (panmictic or expected heterozygosity) for each population sample, whereas Hs is the average of hs values for all population samples. Ht is the heterozygosity that would be observed if all population samples would come from a single population.
(DOC)

Table S9 **Difference (%) in the population-genetics parameters estimated with 97 or 46 ISSR markers.** Note that hs values are estimates of genetic diversity (panmictic or expected heterozygosity) for each population sample, whereas Hs is the average of hs values for all population samples. Ht is the heterozygosity that would be observed if all population samples would come from a single population.
(DOC)

Table S10 **Proportion of individuals from each population included into each group by Structure for 87 loci and K = 2.**
(DOC)

Table S11 **Proportion of individuals from each population included into each group by Structure for 46 loci with K = 2.**
(DOC)

Table S12 **Primers employed and number of markers obtained.**
(DOC)

Table S13 **Summary of crosses and primers tested.**
(DOC)

Table S14 **Expected proportion of marker-carrying progeny for autosomal markers. 1 =  marker presence; 0 =  marker absence.**
(DOC)

Table S15 **Expected proportion of marker-carrying progeny for sex-linked markers. 1 =  marker presence; 0 =  marker absence.**
(DOC)
