# Supplementary figures and images for: Population Genetic Structure of the Grasshopper Eyprepocnemis plorans in the South and East of the Iberian Peninsula
Source: PLoS One. 2013 Mar 8;8(3):e59041. doi: 10.1371/journal.pone.0059041 (PMC3592831; doi:10.1371/journal.pone.0059041)

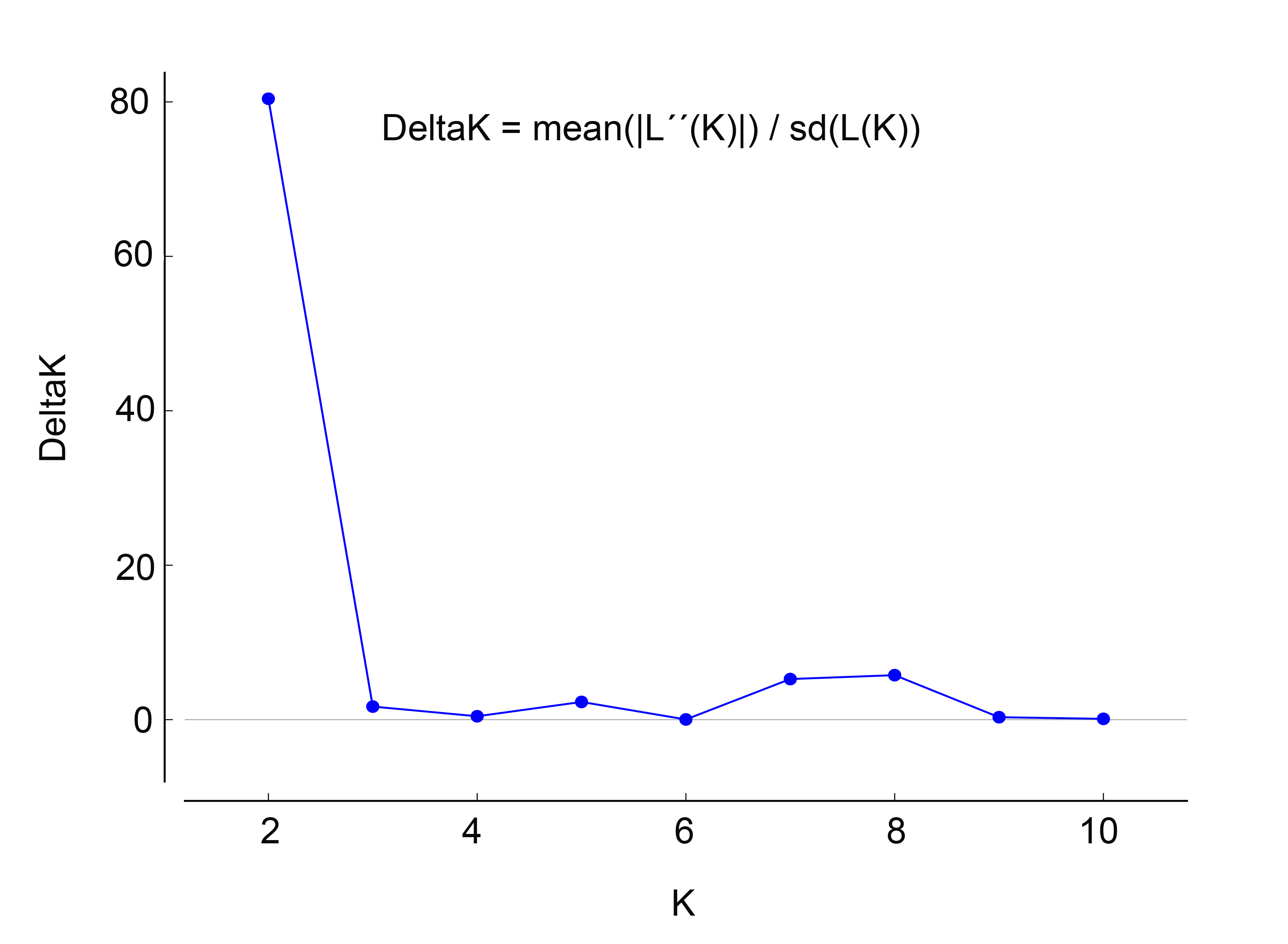

Supplement: Figure S1 — Delta K values with respect to K, according to the calculation method by Evanno et al. [42] . These results were found using the 87 ISSR markers showing no (or low level of) allelic dropout. (TIF) [file pone.0059041.s001.tif]

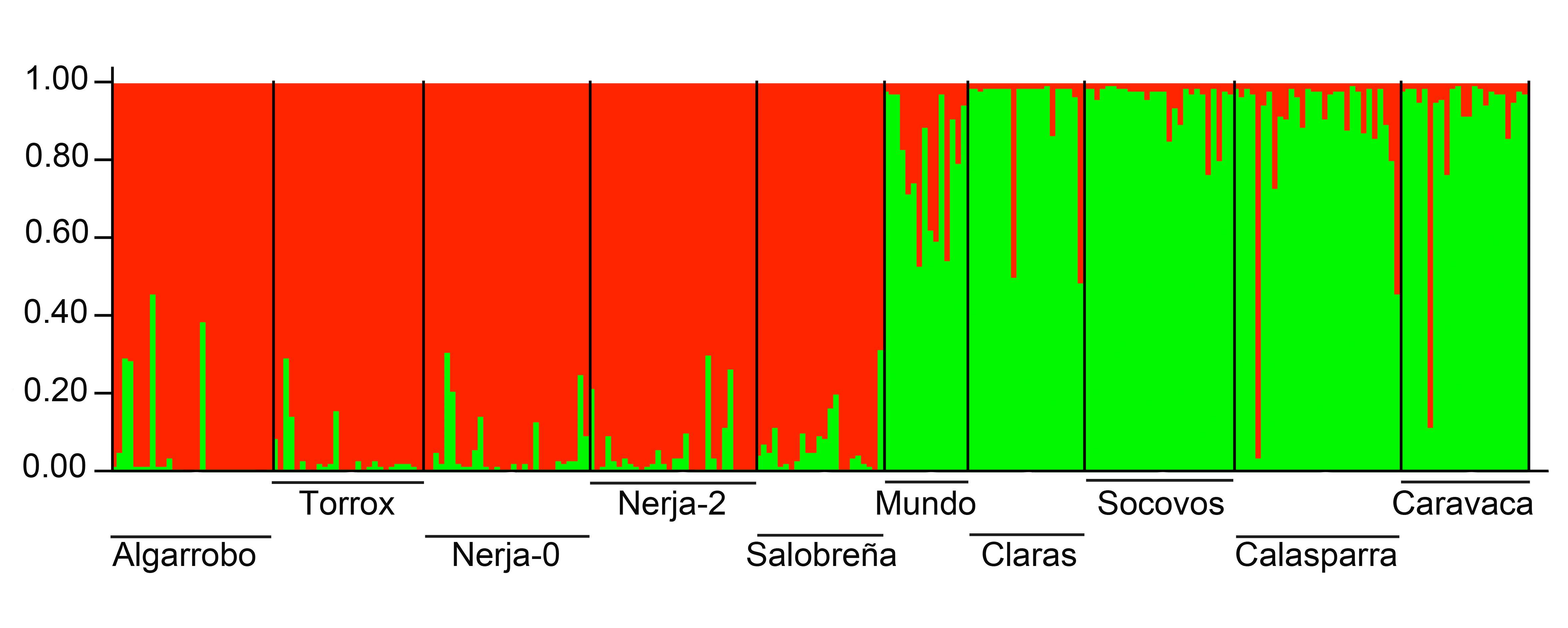

Supplement: Figure S2 — Ancestry of every individual in either of the two groups, using the 87 ISSR markers showing no allelic dropout (or low level thereof), yielded by the Structure software. Each vertical bar represents one of the 255 individuals analyzed. Group 1 (southern region) is represented in red color, and Group 2 (eastern region) is shown in green color. Bar length is proportional to the inferred ancestry values into each group for each individual. (TIF) [file pone.0059041.s002.tif]

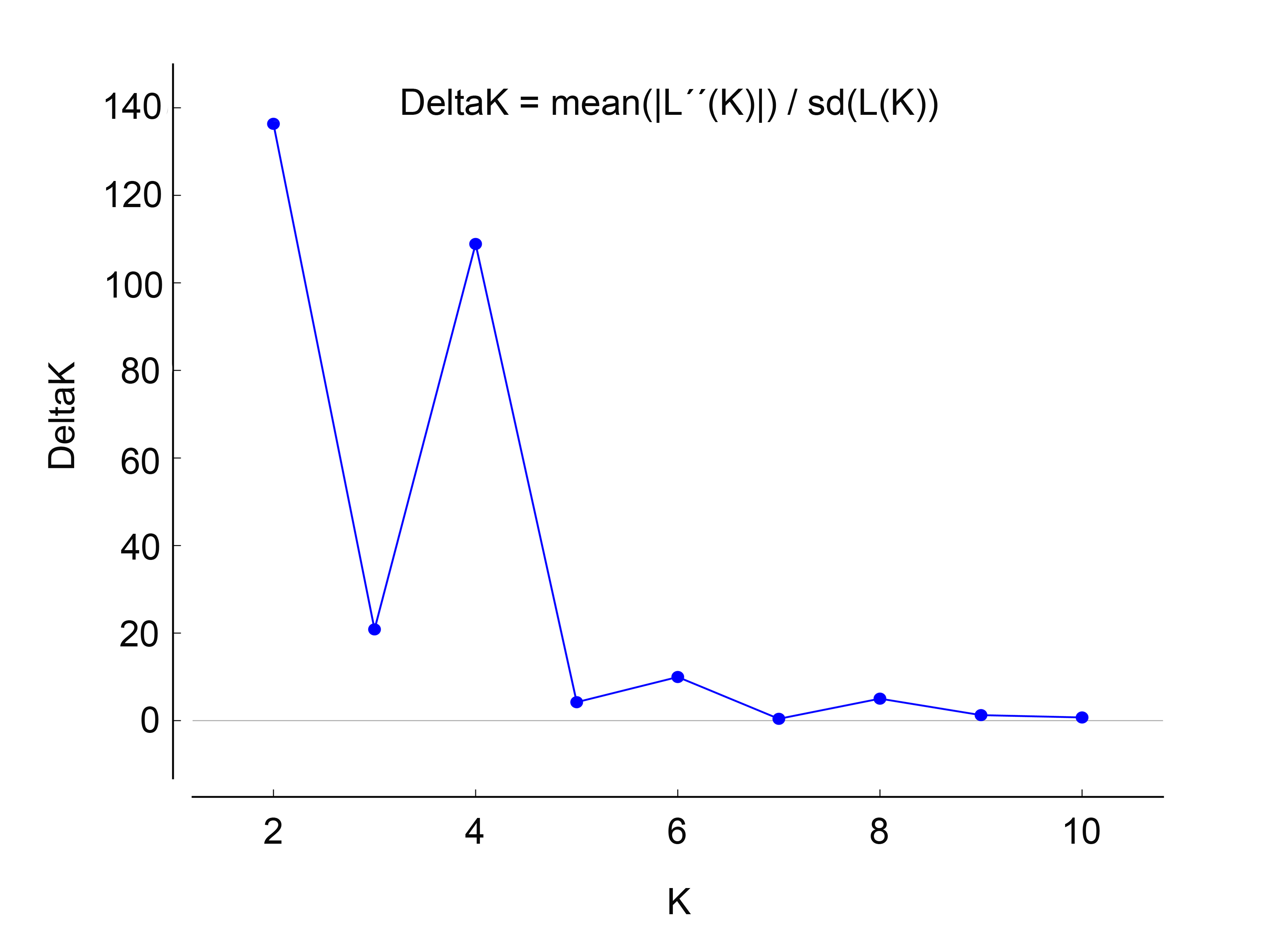

Supplement: Figure S3 — Delta K values with respect to K, according to the calculation method by Evanno et al. [42] . These results were found using the 46 ISSR markers showing no allelic dropout. (TIF) [file pone.0059041.s003.tif]

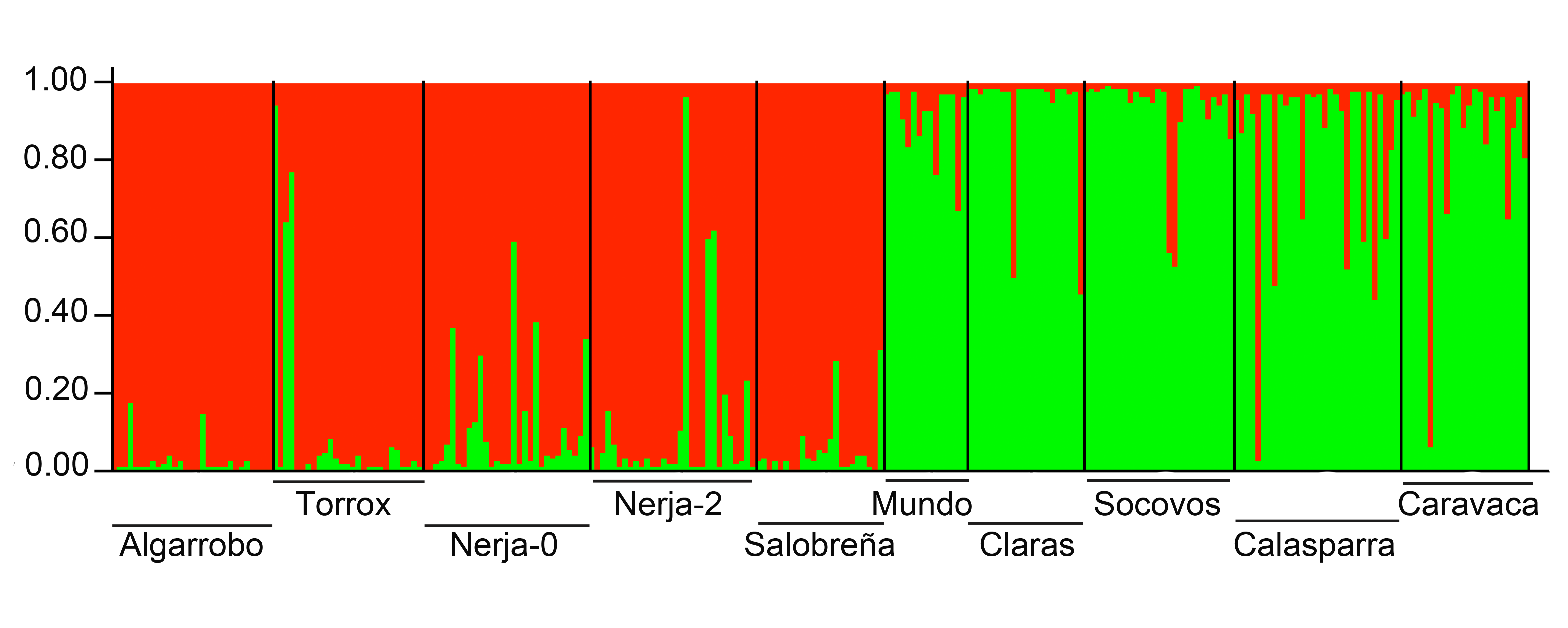

Supplement: Figure S4 — Ancestry of each individual to either of the two groups, using the 46 ISSR markers showing no allelic dropout, yielded by the Structure software. Each vertical bar represents one of the 255 individuals analyzed. Group 1 is represented in red, and group 2 in green color. Bar length is proportional to the inferred ancestry values into each group for each individual. (TIF) [file pone.0059041.s004.tif]
