# Supplementary material for: Population Genetic Structure of the Grasshopper Eyprepocnemis plorans in the South and East of the Iberian Peninsula
Source: PLoS One. 2013 Mar 8;8(3):e59041. doi: 10.1371/journal.pone.0059041 (PMC3592831; doi:10.1371/journal.pone.0059041)
Supplement: Table S1 — Statistical parameters for the different models after Bayesian inference. (DOC) [file pone.0059041.s005.doc]

| **Table S1 Statistical parameters for the different models after Bayesian inference** | | | | |
| --- | --- | --- | --- | --- |
| Model | Dbar | Dhat | pD | DIC |
| *Full* | 3089.56 | 2487.02 | 602.54 | 3692.1 |
| *f=0* | 3093.18 | 2471 | 622.182 | 3715.36 |
| *theta=0* | 6494.25 | 6401.8 | 92.4479 | 6586.69 |
| *f free* | 3222.23 | 2494.27 | 727.964 | 3950.2 |
