# Supplementary material for: Population Genetic Structure of the Grasshopper Eyprepocnemis plorans in the South and East of the Iberian Peninsula
Source: PLoS One. 2013 Mar 8;8(3):e59041. doi: 10.1371/journal.pone.0059041 (PMC3592831; doi:10.1371/journal.pone.0059041)
Supplement: Table S2 — Analysis of Molecular Variance (AMOVA). (DOC) [file pone.0059041.s006.doc]

| **Table S2 Analysis of Molecular Variance (AMOVA)** | | | | | |
| --- | --- | --- | --- | --- | --- |
|
| Source of variation | df | MSD | Variance | % variance | p |
| Between regions (φ_CT_) | 1(G-1) | 266.144 | 179.953 | 15.05 | <0.001 |
| Among populations within regions (φSC) | 8(P-G) | 304.584 | 114.825 | 9.61 | <10^-4^ |
| Within populations (φST) | 245(N-P) | 2.206.429 | 900.583 | 75.34 | <10^-4^ |
