# Supplementary material for: Population Genetic Structure of the Grasshopper Eyprepocnemis plorans in the South and East of the Iberian Peninsula
Source: PLoS One. 2013 Mar 8;8(3):e59041. doi: 10.1371/journal.pone.0059041 (PMC3592831; doi:10.1371/journal.pone.0059041)
Supplement: Table S3 — Likelihood values obtained for calculation of the number of groups (K) best fitting the data through the Evanno method. Reps = replicates. (DOC) [file pone.0059041.s007.doc]

| **Table S3 Likelihood values obtained for calculation of the number of groups (K) best fitting the data through the Evanno method. Reps= replicates** | | | | | | |
| --- | --- | --- | --- | --- | --- | --- |
| K | Reps | Mean LnP(K) | Stdev LnP(K) | Ln'(K) | |Ln''(K)| | Delta K |
| 1 | 10 | -13852.85 | 1.23 | — | — | — |
| 2 | 10 | -12895.8 | 7.48 | 957.05 | 1183.62 | 158.21 |
| 3 | 10 | -13122.37 | 937.42 | -226.57 | 842.20 | 0.90 |
| 4 | 10 | -12506.74 | 241.20 | 615.63 | 313.85 | 1.30 |
| 5 | 10 | -12204.96 | 30.96 | 301.78 | 112.50 | 3.63 |
| 6 | 10 | -12015.68 | 46.79 | 189.28 | 14.22 | 0.30 |
| 7 | 10 | -11840.62 | 36.95 | 175.06 | 78.00 | 2.11 |
| 8 | 10 | -11743.56 | 85.71 | 97.06 | 5.29 | 0.06 |
| 9 | 10 | -11641.21 | 10.89 | 102.35 | 244.77 | 22.48 |
| 10 | 10 | -11783.63 | 180.99 | -142.42 | 71.30 | 0.39 |
| 11 | 10 | -11997.35 | 498.00 | -213.72 | — | — |
