# Supplementary material for: Population Genetic Structure of the Grasshopper Eyprepocnemis plorans in the South and East of the Iberian Peninsula
Source: PLoS One. 2013 Mar 8;8(3):e59041. doi: 10.1371/journal.pone.0059041 (PMC3592831; doi:10.1371/journal.pone.0059041)
Supplement: Table S4 — Pairwise-Fst genetic distances (bottom) and geographical distances in Km (top). (DOC) [file pone.0059041.s008.doc]

| **Table S4 Pairwise-Fst genetic distances (bottom) and geographical distances in Km (top)** | | | | | | | | | | |
| --- | --- | --- | --- | --- | --- | --- | --- | --- | --- | --- |
|  | Algarrobo | Torrox | Nerja-0 | Nerja-2 | Salobreña | Mundo | Claras | Socovos | Calasparra | Caravaca |
| Algarrobo |  | 8.5 | 13.2 | 17.1 | 40.5 | 238.3 | 247.3 | 253.8 | 275.6 | 266.5 |
| Torrox | 0.0432 |  | 4.8 | 8.9 | 32.1 | 233.6 | 241.4 | 248.6 | 270.5 | 260.8 |
| Nerja-0 | 0.032 | 0.0276 |  | 4.1 | 27.4 | 229.9 | 237.2 | 244.8 | 266.7 | 256.7 |
| Nerja-2 | 0.0501 | 0.0243 | 0.0203 |  | 23.6 | 226.4 | 233.5 | 241.2 | 263.1 | 252.9 |
| Salobreña | 0.0814 | 0.0884 | 0.0888 | 0.1136 |  | 213.8 | 217.5 | 227.1 | 248.9 | 248.9 |
| Mundo | 0.1455 | 0.1232 | 0.1414 | 0.1396 | 0.1492 |  | 40.49 | 21.9 | 25.5 | 40 |
| Claras | 0.1921 | 0.168 | 0.1845 | 0.1744 | 0.1838 | 0.0698 |  | 21.9 | 46.6 | 41.4 |
| Socovos | 0.1737 | 0.1405 | 0.1573 | 0.132 | 0.1786 | 0.0641 | 0.041 |  | 25.4 | 26.9 |
| Calasparra | 0.1646 | 0.1427 | 0.158 | 0.1489 | 0.1593 | 0.0381 | 0.0701 | 0.0555 |  | 20 |
| Caravaca | 0.1882 | 0.1604 | 0.1753 | 0.1478 | 0.1899 | 0.1426 | 0.0746 | 0.0418 | 0.1365 |  |
