# Supplementary material for: Population Genetic Structure of the Grasshopper Eyprepocnemis plorans in the South and East of the Iberian Peninsula
Source: PLoS One. 2013 Mar 8;8(3):e59041. doi: 10.1371/journal.pone.0059041 (PMC3592831; doi:10.1371/journal.pone.0059041)
Supplement: Table S5 — Dice's dissimilarity coefficients between populations (bottom) and standard errors (top). (DOC) [file pone.0059041.s009.doc]

| **Table S5 Dice's dissimilarity coefficients between populations (bottom) and standard errors (top)** | | | | | | | | | | |
| --- | --- | --- | --- | --- | --- | --- | --- | --- | --- | --- |
|  | Algarrobo | Torrox | Nerja-0 | Nerja-2 | Salobreña | Mundo | Claras | Socovos | Calasparra | Caravaca |
| Algarrobo |  | 0.003 | 0.003 | 0.003 | 0.004 | 0.004 | 0.003 | 0.004 | 0.003 | 0.004 |
| Torrox | 0.398 |  | 0.003 | 0.004 | 0.003 | 0.004 | 0.003 | 0.004 | 0.003 | 0.004 |
| Nerja-0 | 0.378 | 0.382 |  | 0.003 | 0.003 | 0.004 | 0.003 | 0.004 | 0.003 | 0.003 |
| Nerja-2 | 0.406 | 0.415 | 0.381 |  | 0.003 | 0.004 | 0.003 | 0.004 | 0.003 | 0.003 |
| Salobreña | 0.408 | 0.424 | 0.408 | 0.443 |  | 0.004 | 0.004 | 0.004 | 0.004 | 0.004 |
| Mundo | 0.453 | 0.458 | 0.451 | 0.472 | 0.453 |  | 0.005 | 0.006 | 0.004 | 0.005 |
| Claras | 0.515 | 0.517 | 0.506 | 0.529 | 0.522 | 0.436 |  | 0.005 | 0.004 | 0.004 |
| Socovos | 0.55 | 0.552 | 0.539 | 0.549 | 0.564 | 0.475 | 0.48 |  | 0.005 | 0.005 |
| Calasparra | 0.477 | 0.491 | 0.476 | 0.501 | 0.466 | 0.39 | 0.434 | 0.473 |  | 0.004 |
| Caravaca | 0.552 | 0.557 | 0.544 | 0.564 | 0.57 | 0.55 | 0.488 | 0.536 | 0.537 |  |
