# Supplementary material for: Population Genetic Structure of the Grasshopper Eyprepocnemis plorans in the South and East of the Iberian Peninsula
Source: PLoS One. 2013 Mar 8;8(3):e59041. doi: 10.1371/journal.pone.0059041 (PMC3592831; doi:10.1371/journal.pone.0059041)
Supplement: Table S6 — Failure rate of PCR amplification for every marker (%) under the two controls performed. Dropout values above 30% are marked in bold. N1 and N2 indicate the number of individuals where a given allele was tested for PCR repeatability under controls 1 and 2, respectively. (DOC) [file pone.0059041.s010.doc]

| **Table S6 Failure rate of PCR amplification for every marker (%) under the two controls performed. Dropout values above 30% are marked in bold. N1 and N2 indicate the number of individuals where a given allele was tested for PCR repeatability under controls 1 and 2, respectively** | | | | | |
| --- | --- | --- | --- | --- | --- |
| Allele | Size (bp) | Control 1 | N1 | Control 2 | N2 |
| 6-250 | 250 | 0 | 9 | 0 | 6 |
| 6-330 | 330 | 0 | 9 | 0 | 6 |
| 6-350 | 350 | 0 | 9 | 0 | 6 |
| 6-380 | 380 | 0 | 9 | 0 | 6 |
| 6-450 | 450 | 0 | 9 | 16.67 | 6 |
| 6-500 | 500 | 0 | 9 | 0 | 6 |
| 6-550 | 550 | 0 | 9 | 0 | 6 |
| 6-630 | 630 | 0 | 9 | 0 | 6 |
| 6-750 | 750 | 0 | 9 | 0 | 6 |
| 6-800 | 800 | 0 | 9 | 0 | 6 |
| 6-900 | 900 | 0 | 9 | 0 | 6 |
| 6-1000 | 1000 | 0 | 9 | 0 | 6 |
| 6-1200 | 1200 | 0 | 9 | 0 | 6 |
| 6-1300 | 1300 | 0 | 9 | 0 | 6 |
| 6-1400 | 1400 | 0 | 9 | 0 | 6 |
| 6-1600 | 1600 | 0 | 9 | 0 | 6 |
| 7-250 | 250 | 0 | 8 | 0 | 14 |
| 7-320 | 320 | 0 | 8 | 21.43 | 14 |
| 7-350 | 350 | 0 | 8 | 0 | 14 |
| 7-380 | 380 | 0 | 8 | 21.43 | 14 |
| 7-430 | 430 | 0 | 8 | 7.14 | 14 |
| 7-480 | 480 | 0 | 8 | 0 | 14 |
| 7-550 | 550 | 0 | 8 | 7.14 | 14 |
| 7-600 | 600 | 0 | 8 | 21.43 | 14 |
| 7-650 | 650 | 0 | 8 | 0 | 14 |
| 7-700 | 700 | 0 | 8 | 28.57 | 14 |
| 7-800 | 800 | 0 | 8 | 21.43 | 14 |
| 7-900 | 900 | 0 | 8 | 7.14 | 14 |
| 7-1000 | 1000 | 0 | 8 | 14.29 | 14 |
| 7-1100 | 1100 | 0 | 8 | 28.57 | 14 |
| 7-1150 | 1150 | 0 | 8 | 7.14 | 14 |
| 7-1400 | 1400 | 0 | 8 | 21.43 | 14 |
| 7-1600 | 1600 | 0 | 8 | 7.14 | 14 |
| 7-1800 | 1800 | 0 | 8 | 0 | 14 |
| 7->2000 | >2000 | 0 | 8 | 0 | 14 |
| 14-300 | 300 | 0 | 8 | 11.11 | 9 |
| 14-400 | 400 | 0 | 8 | 0 | 9 |
| 14-500 | 500 | 12.5 | 8 | 0 | 9 |
| 14-600 | 600 | 0 | 8 | 0 | 9 |
| 14-750 | 750 | 0 | 8 | 11.11 | 9 |
| 14-800 | 800 | 0 | 8 | 0 | 9 |
| 14-850 | 850 | 0 | 8 | 11.11 | 9 |
| 14-1250 | 1250 | 0 | 8 | 11.11 | 9 |
| 14-1400 | 1400 | 12.5 | 8 | 0 | 9 |
| 14-1450 | 1450 | 0 | 8 | 0 | 9 |
| 14-1800 | 1800 | 0 | 8 | 11.11 | 9 |
| 26-280 | 280 | 0 | 7 | 0 | 7 |
| 26-300 | 300 | 0 | 7 | 14.29 | 7 |
| 26-340 | 340 | 0 | 7 | 0 | 7 |
| 26-380 | 380 | 14.29 | 7 | 28.57 | 7 |
| 26-430 | 430 | 0 | 7 | 0 | 7 |
| 26-480 | 480 | 0 | 7 | 14.29 | 7 |
| 26-500 | 500 | **42.86** | 7 | **42.86** | 7 |
| 26-550 | 550 | 0 | 7 | 14.29 | 7 |
| 26-600 | 600 | 28.57 | 7 | 14.29 | 7 |
| 26-650 | 650 | 0 | 7 | **42.86** | 7 |
| 26-700 | 700 | 0 | 7 | 28.57 | 7 |
| 26-750 | 750 | 0 | 7 | **42.86** | 7 |
| 26-800 | 800 | 14.29 | 7 | 14.29 | 7 |
| 26-900 | 900 | 0 | 7 | 28.57 | 7 |
| 26-1000 | 1000 | 0 | 7 | **42.86** | 7 |
| 26-1100 | 1100 | 0 | 7 | 0 | 7 |
| 26-1200 | 1200 | 0 | 7 | 0 | 7 |
| 26-1300 | 1300 | 0 | 7 | 0 | 7 |
| 26-1600 | 1600 | 0 | 7 | 0 | 7 |
| 26-1800 | 1800 | 0 | 7 | 0 | 7 |
| 26-2000 | 2000 | 0 | 7 | 0 | 7 |
| 26>2000 | 26>2000 | 0 | 7 | 14.29 | 7 |
| 39-180 | 180 | 0 | 5 | 0 | 7 |
| 39-200 | 200 | 0 | 5 | 0 | 7 |
| 39-220 | 220 | 0 | 5 | 28.57 | 7 |
| 39-250 | 250 | 0 | 5 | **42.86** | 7 |
| 39-280 | 280 | 0 | 5 | 0 | 7 |
| 39-320 | 320 | 0 | 5 | 0 | 7 |
| 39-390 | 390 | 0 | 5 | 0 | 7 |
| 39-410 | 410 | 0 | 5 | 0 | 7 |
| 39-450 | 450 | 0 | 5 | 0 | 7 |
| 39-500 | 500 | 0 | 5 | 0 | 7 |
| 39-550 | 550 | 0 | 5 | 0 | 7 |
| 39-600 | 600 | 0 | 5 | 0 | 7 |
| 39-650 | 650 | 0 | 5 | 0 | 7 |
| 39-700 | 700 | 0 | 5 | 0 | 7 |
| 39-800 | 800 | 0 | 5 | 14.29 | 7 |
| 39-880 | 880 | 0 | 5 | 14.29 | 7 |
| 39-1000 | 1000 | 0 | 5 | 0 | 7 |
| 39-1100 | 1100 | 0 | 5 | 0 | 7 |
| 43-250 | 250 | 0 | 6 | 18.18 | 11 |
| 43-280 | 280 | 0 | 6 | 18.18 | 11 |
| 43-320 | 320 | 0 | 6 | 9.09 | 11 |
| 43-380 | 380 | 0 | 6 | 9.09 | 11 |
| 43-400 | 400 | 0 | 6 | 27.27 | 11 |
| 43-490 | 490 | 0 | 6 | 0 | 11 |
| 43-540 | 540 | 0 | 6 | **54.55** | 11 |
| 43-600 | 600 | 0 | 6 | 9.09 | 11 |
| 43-650 | 650 | 0 | 6 | 18.18 | 11 |
| 43-780 | 780 | 0 | 6 | 0 | 11 |
| 43-800 | 800 | 16.67 | 6 | 0 | 11 |
