# Supplementary material for: Population Genetic Structure of the Grasshopper Eyprepocnemis plorans in the South and East of the Iberian Peninsula
Source: PLoS One. 2013 Mar 8;8(3):e59041. doi: 10.1371/journal.pone.0059041 (PMC3592831; doi:10.1371/journal.pone.0059041)
Supplement: Table S7 — Inheritance analysis of ISSR markers by means of the goodness-of-fit chi square test. 0 = Absence of the allele, 1 = Presence of the allele, Obs.1 = Observed frequency of 1, exp.1 = Expected frequency of 1. Progeny analyses showing significance after sequential Bonferroni are indicated by an asterisk. (DOC) [file pone.0059041.s011.doc]

| **Table S7 Inheritance analysis of ISSR markers by means of the goodness-of-fit chi square test. 0= Absence of the allele, 1= Presence of the allele, Obs.1= Observed frequency of 1, exp.1= Expected frequency of 1. Progeny analyses showing significance after sequential Bonferroni are indicated by an asterisk** | | | | | | | | | | | | | |
| --- | --- | --- | --- | --- | --- | --- | --- | --- | --- | --- | --- | --- | --- |
|  |  |  |  |  | Progeny | | | | | | | |  |
| ISSR |  | Parents | |  | Observed | | | |  | Expected | | |  |
| Allele | Cross | ♀ | ♂ |  | 0 | 1 | Total | obs.1 |  | exp.1 | 0 | 1 | χ^2^ (p) |
| 6-350 | ♀ 4 x ♂ 19 | 1 | 1 |  | 4 | 24 | 28 | 0.857 |  | 0.75 | 7 | 21 | 1.714 (0.190) |
| 6-500 | ♀ 4 x ♂ 19 | 1 | 1 |  | 6 | 22 | 28 | 0.786 |  | 0.75 | 7 | 21 | 0.190 (0.663) |
| 6-1300 | ♀ 4 x ♂ 19 | 1 | 0 |  | 21 | 7 | 28 | 0.250 |  | 0.5 | 14 | 14 | 7 (0.008) |
| 7-350 | ♀ 4 x ♂ 19 | 1 | 0 |  | 16 | 12 | 28 | 0.429 |  | 0.5 | 14 | 14 | 0.571 (0.450) |
| 7-430 | ♀ 19 x ♂ 27 | 1 | 0 |  | 8 | 10 | 18 | 0.556 |  | 0.5 | 9 | 9 | 0.222 (0.637) |
|  | ♀ 24 x ♂ 32 | 1 | 1 |  | 1 | 14 | 15 | 0.933 |  | 0.75 | 3.75 | 11.25 | 2.689 (0.101) |
|  | ♀ 4 x ♂ 19 | 1 | 1 |  | 0 | 28 | 28 | 1 |  |  |  |  |  |
|  | ♀ 12 x ♂ 3 | 1 | 1 |  | 2 | 12 | 14 | 0.857 |  | 0.75 | 3.5 | 10.5 | 0.857 (0.3555) |
|  | ♀ 29 x ♂ 3 | 1 | 1 |  | 4 | 6 | 10 | 0.6 |  | 0.75 | 2.5 | 7.5 | 1.2 (0.273) |
|  | ♀ 3 x ♂ 3 | 1 | 1 |  | 0 | 20 | 20 | 1 |  |  |  |  |  |
|  | ♀ 5 x ♂ 7 | 1 | 1 |  | 0 | 16 | 16 | 1 |  |  |  |  |  |
| 7-480 | ♀ 4 x ♂ 19 | 0 | 1 |  | 11 | 17 | 28 | 0.607 |  | 0.5 | 14 | 14 | 1.286 (0.257) |
|  | ♀ 12 x ♂ 3 | 1 | 0 |  | 9 | 5 | 14 | 0.357 |  | 0.5 | 7 | 7 | 1.143 (0.285) |
| 7-600 | ♀ 19 x ♂ 27 | 0 | 1 |  | 8 | 10 | 18 | 0.556 |  | 0.5 | 9 | 9 | 0.222 (0.637) |
|  | ♀ 24 x ♂ 32 | 1 | 1 |  | 4 | 11 | 15 | 0.733 |  | 0.75 | 3.75 | 11.25 | 0.022 (0.881) |
|  | ♀ 4 x ♂ 19 | 1 | 1 |  | 10 | 18 | 28 | 0.643 |  | 0.75 | 7 | 21 | 1.714 (0.190) |
|  | ♀ 12 x ♂ 3 | 1 | 1 |  | 2 | 12 | 14 | 0.857 |  | 0.75 | 3.5 | 10.5 | 0.857 (0.355) |
|  | ♀ 29 x ♂ 3 | 0 | 1 |  | 3 | 7 | 10 | 0.7 |  | 0.5 | 5 | 5 | 1.6 (0.206) |
|  | ♀ 3 x ♂ 3 | 0 | 1 |  | 8 | 12 | 20 | 0.6 |  | 0.5 | 10 | 10 | 0.8 (0.371) |
|  | ♀ 5 x ♂ 7 | 0 | 1 |  | 13 | 3 | 16 | 0.188 |  | 0.5 | 8 | 8 | 6.25 (0.012) |
| 7-700 | ♀ 4 x ♂ 19 | 1 | 1 |  | 5 | 23 | 28 | 0.821 |  | 0.75 | 7 | 21 | 0.762 (0.383) |
|  | ♀ 12 x ♂ 3 | 1 | 0 |  | 8 | 6 | 14 | 0.429 |  | 0.5 | 7 | 7 | 0.286 (0.593) |
| 7-800 | ♀ 19 x ♂ 27 | 1 | 1 |  | 2 | 16 | 18 | 0.889 |  | 0.75 | 4.5 | 13.5 | 1.852 (0.174) |
|  | ♀ 24 x ♂ 32 | 1 | 1 |  | 0 | 15 | 15 | 1 |  |  |  |  |  |
|  | ♀ 4 x ♂ 19 | 1 | 0 |  | 0 | 28 | 28 | 1 |  |  |  |  |  |
|  | ♀ 12 x ♂ 3 | 1 | 0 |  | 0 | 14 | 14 | 1 |  |  |  |  |  |
|  | ♀ 29 x ♂ 3 | 1 | 0 |  | 0 | 10 | 10 | 1 |  |  |  |  |  |
|  | ♀ 3 x ♂ 3 | 1 | 1 |  | 1 | 19 | 20 | 0.95 |  | 0.75 | 5 | 15 | 4.267 (0.039) |
|  | ♀ 5 x ♂ 7 | 1 | 1 |  | 0 | 16 | 16 | 1 |  |  |  |  |  |
| 7-1000 | ♀ 19 x ♂ 27 | 0 | 1 |  | 16 | 2 | 18 | 0.111 |  | 0.5 | 9 | 9 | 10.889 (0.001) |
|  | ♀ 24 x ♂ 32 | 0 | 1 |  | 6 | 9 | 15 | 0.6 |  | 0.5 | 7.5 | 7.5 | 0.6 (0.439) |
|  | ♀ 4 x ♂ 19 | 1 | 1 |  | 18 | 10 | 28 | 0.357 |  | 0.75 | 7 | 21 | 23.048 (1.580E-06)* |
|  | ♀ 12 x ♂ 3 | 1 | 0 |  | 8 | 6 | 14 | 0.429 |  | 0.5 | 7 | 7 | 0.286 (0.593) |
|  | ♀ 3 x ♂ 3 | 1 | 1 |  | 6 | 14 | 20 | 0.7 |  | 0.75 | 5 | 15 | 0.267 (0.606) |
|  | ♀ 5 x ♂ 7 | 0 | 1 |  | 11 | 5 | 16 | 0.313 |  | 0.5 | 8 | 8 | 2.25 (0.134) |
| 7-1100 | ♀ 19 x ♂ 27 | 1 | 1 |  | 15 | 3 | 18 | 0.167 |  | 0.75 | 4.5 | 13.5 | 32.667 (1.09E-08)* |
|  | ♀ 24 x ♂ 32 | 1 | 0 |  | 6 | 9 | 15 | 0.6 |  | 0.5 | 7.5 | 7.5 | 0.6 (0.439) |
|  | ♀ 4 x ♂ 19 | 1 | 1 |  | 15 | 13 | 28 | 0.464 |  | 0.75 | 7 | 21 | 12.190 (0.000)* |
|  | ♀ 12 x ♂ 3 | 1 | 0 |  | 7 | 7 | 14 | 0.5 |  | 0.5 | 7 | 7 | 0 (1) |
|  | ♀ 5 x ♂ 7 | 0 | 1 |  | 3 | 13 | 16 | 0.813 |  | 0.5 | 8 | 8 | 6.25 (0.012) |
| 7-1400 | ♀ 19 x ♂ 27 | 1 | 0 |  | 12 | 6 | 18 | 0.333 |  | 0.5 | 9 | 9 | 2 (0.157) |
|  | ♀ 24 x ♂ 32 | 0 | 1 |  | 11 | 4 | 15 | 0.267 |  | 0.5 | 7.5 | 7.5 | 3.267 (0.071) |
|  | ♀ 4 x ♂ 19 | 0 | 1 |  | 23 | 5 | 28 | 0.179 |  | 0.5 | 14 | 14 | 11.571 (0.001) |
|  | ♀ 12 x ♂ 3 | 1 | 0 |  | 12 | 2 | 14 | 0.143 |  | 0.5 | 7 | 7 | 7.143 (0.008) |
|  | ♀ 29 x ♂ 3 | 1 | 0 |  | 6 | 4 | 10 | 0.4 |  | 0.5 | 5 | 5 | 0.4 (0.527) |
|  | ♀ 3 x ♂ 3 | 1 | 1 |  | 11 | 9 | 20 | 0.45 |  | 0.75 | 5 | 15 | 9.6 (0.002) |
| 7-1600 | ♀ 24 x ♂ 32 | 1 | 0 |  | 10 | 5 | 15 | 0.333 |  | 0.5 | 7.5 | 7.5 | 1.667 (0.197) |
|  | ♀ 12 x ♂ 3 | 1 | 0 |  | 7 | 7 | 14 | 0.5 |  | 0.5 | 7 | 7 | 0 (1) |
|  | ♀ 3 x ♂ 3 | 1 | 1 |  | 10 | 10 | 20 | 0.5 |  | 0.75 | 5 | 15 | 6.667 (0.010) |
|  | ♀ 5 x ♂ 7 | 1 | 0 |  | 7 | 9 | 16 | 0.563 |  | 0.5 | 8 | 8 | 0.25 (0.617) |
| 14-400 | ♀ 4 x ♂ 19 | 1 | 1 |  | 11 | 13 | 24 | 0.542 |  | 0.75 | 6 | 18 | 5.556 (0.018) |
| 14-500 | ♀ 19 x ♂ 27 | 1 | 0 |  | 10 | 5 | 15 | 0.333 |  | 0.5 | 7.5 | 7.5 | 1.667 (0.197) |
|  | ♀ 4 x ♂ 19 | 1 | 1 |  | 11 | 13 | 24 | 0.542 |  | 0.75 | 6 | 18 | 5.556 (0.018) |
|  | ♀ 2 x ♂ 4 | 0 | 1 |  | 12 | 9 | 21 | 0.429 |  | 0.5 | 10.5 | 10.5 | 0.429 (0.513) |
|  | ♀ 5 x ♂ 7 | 1 | 0 |  | 9 | 8 | 17 | 0.471 |  | 0.5 | 8.5 | 8.5 | 0.059 (0.808) |
| 14-600 | ♀ 19 x ♂ 27 | 1 | 1 |  | 0 | 15 | 15 | 1 |  |  |  |  |  |
|  | ♀ 4 x ♂ 19 | 1 | 1 |  | 6 | 18 | 24 | 0.75 |  | 0.75 | 6 | 18 | 0 (1) |
|  | ♀ 12 x ♂ 3 | 1 | 0 |  | 9 | 3 | 12 | 0.25 |  | 0.5 | 6 | 6 | 3 (0.083) |
|  | ♀ 13 x ♂ 20 | 1 | 1 |  | 2 | 26 | 28 | 0.929 |  | 0.75 | 7 | 21 | 4.762 (0.029) |
|  | ♀ 16 x ♂ 14 | 1 | 1 |  | 8 | 9 | 17 | 0.529 |  | 0.75 | 4.25 | 12.75 | 4.412 (0.036) |
|  | ♀ 2 x ♂ 4 | 1 | 1 |  | 0 | 21 | 21 | 1 |  |  |  |  |  |
|  | ♀ 5 x ♂ 7 | 0 | 1 |  | 2 | 15 | 17 | 0.882 |  | 0.5 | 8.5 | 8.5 | 9.941 (0.002) |
| 14-750 | ♀ 19 x ♂ 27 | 1 | 0 |  | 13 | 2 | 15 | 0.133 |  | 0.5 | 7.5 | 7.5 | 8.067 (0.005) |
|  | ♀ 4 x ♂ 19 | 0 | 1 |  | 13 | 11 | 24 | 0.458 |  | 0.5 | 12 | 12 | 0.167 (0.683) |
|  | ♀ 12 x ♂ 3 | 1 | 0 |  | 5 | 7 | 12 | 0.583 |  | 0.5 | 6 | 6 | 0.333 (0.564) |
|  | ♀ 13 x ♂ 20 | 1 | 1 |  | 19 | 9 | 28 | 0.321 |  | 0.75 | 7 | 21 | 27.429 (1.62E-07)* |
|  | ♀ 16 x ♂ 14 | 0 | 1 |  | 10 | 7 | 17 | 0.412 |  | 0.5 | 8.5 | 8.5 | 0.529 (0.467) |
|  | ♀ 2 x ♂ 4 | 1 | 1 |  | 5 | 16 | 21 | 0.762 |  | 0.75 | 5.25 | 15.75 | 0.016 (0.900) |
|  | ♀ 5 x ♂ 7 | 0 | 1 |  | 3 | 14 | 17 | 0.824 |  | 0.5 | 8.5 | 8.5 | 7.118 (0.008) |
| 14-800 | ♀ 19 x ♂ 27 | 1 | 1 |  | 1 | 14 | 15 | 0.933 |  | 0.75 | 3.75 | 11.25 | 2.689 (0.101) |
|  | ♀ 4 x ♂ 19 | 1 | 0 |  | 12 | 12 | 24 | 0.5 |  | 0.5 | 12 | 12 | 0 (1) |
|  | ♀ 12 x ♂ 3 | 1 | 1 |  | 4 | 8 | 12 | 0.667 |  | 0.75 | 3 | 9 | 0.444 (0.505) |
|  | ♀ 13 x ♂ 20 | 1 | 0 |  | 12 | 16 | 28 | 0.571 |  | 0.5 | 14 | 14 | 0.571 (0.450) |
|  | ♀ 16 x ♂ 14 | 1 | 1 |  | 4 | 13 | 17 | 0.765 |  | 0.75 | 4.25 | 12.75 | 0.020 (0.889) |
|  | ♀ 2 x ♂ 4 | 1 | 0 |  | 4 | 17 | 21 | 0.810 |  | 0.5 | 10.5 | 10.5 | 8.048 (0.005) |
|  | ♀ 5 x ♂ 7 | 1 | 0 |  | 11 | 6 | 17 | 0.353 |  | 0.5 | 8.5 | 8.5 | 1.471 (0.225) |
| 14-850 | ♀ 19 x ♂ 27 | 1 | 1 |  | 6 | 9 | 15 | 0.6 |  | 0.75 | 3.75 | 11.25 | 1.8 (0.180) |
|  | ♀ 4 x ♂ 19 | 0 | 1 |  | 10 | 14 | 24 | 0.583 |  | 0.5 | 12 | 12 | 0.667 (0.441) |
|  | ♀ 13 x ♂ 20 | 0 | 1 |  | 20 | 8 | 28 | 0.286 |  | 0.5 | 14 | 14 | 5.143 (0.023) |
|  | ♀ 16 x ♂ 14 | 0 | 1 |  | 11 | 6 | 17 | 0.353 |  | 0.5 | 8.5 | 8.5 | 1.471 (0.225) |
| 14-1250 | ♀ 19 x ♂ 27 | 1 | 1 |  | 0 | 15 | 15 | 1 |  |  |  |  |  |
|  | ♀ 4 x ♂ 19 | 1 | 1 |  | 2 | 22 | 24 | 0.917 |  | 0.75 | 6 | 18 | 3.556 (0.059) |
|  | ♀ 12 x ♂ 3 | 1 | 0 |  | 1 | 11 | 12 | 0.917 |  | 0.5 | 6 | 6 | 8.333 (0.004) |
|  | ♀ 13 x ♂ 20 | 1 | 1 |  | 5 | 23 | 28 | 0.821 |  | 0.75 | 7 | 21 | 0.762 (0.383) |
|  | ♀ 16 x ♂ 14 | 0 | 1 |  | 9 | 8 | 17 | 0.471 |  | 0.5 | 8.5 | 8.5 | 0.059 (0.808) |
|  | ♀ 2 x ♂ 4 | 1 | 1 |  | 3 | 18 | 21 | 0.857 |  | 0.75 | 5.25 | 15.75 | 1.286 (0.257) |
|  | ♀ 5 x ♂ 7 | 0 | 1 |  | 1 | 16 | 17 | 0.941 |  | 0.5 | 8.5 | 8.5 | 13.235 (0)* |
| 14-1400 | ♀ 19 x ♂ 27 | 1 | 1 |  | 3 | 12 | 15 | 0.8 |  | 0.75 | 3.75 | 11.25 | 0.2 (0.655) |
|  | ♀ 4 x ♂ 19 | 1 | 1 |  | 4 | 20 | 24 | 0.833 |  | 0.75 | 6 | 18 | 0.889 (0.346) |
|  | ♀ 12 x ♂ 3 | 1 | 0 |  | 5 | 7 | 12 | 0.583 |  | 0.5 | 6 | 6 | 0.333 (0.564) |
|  | ♀ 13 x ♂ 20 | 1 | 1 |  | 6 | 22 | 28 | 0.786 |  | 0.75 | 7 | 21 | 0.190 (0.663) |
|  | ♀ 16 x ♂ 14 | 0 | 1 |  | 9 | 8 | 17 | 0.471 |  | 0.5 | 8.5 | 8.5 | 0.059 (0.808) |
|  | ♀ 2 x ♂ 4 | 1 | 1 |  | 1 | 20 | 21 | 0.952 |  | 0.75 | 5.25 | 15.75 | 4.587 (0.032) |
|  | ♀ 5 x ♂ 7 | 0 | 1 |  | 3 | 14 | 17 | 0.824 |  | 0.5 | 8.5 | 8.5 | 7.118 (0.008) |
| 14-1450 | ♀ 12 x ♂ 3 | 1 | 0 |  | 5 | 7 | 12 | 0.583 |  | 0.5 | 6 | 6 | 0.333 (0.564) |
|  | ♀ 13 x ♂ 20 | 0 | 1 |  | 19 | 9 | 28 | 0.321 |  | 0.5 | 14 | 14 | 3.571 (0.059) |
|  | ♀ 16 x ♂ 14 | 0 | 1 |  | 15 | 2 | 17 | 0.118 |  | 0.5 | 8.5 | 8.5 | 9.941 (0.002) |
| 14-1800 | ♀ 19 x ♂ 27 | 1 | 0 |  | 6 | 9 | 15 | 0.6 |  | 0.5 | 7.5 | 7.5 | 0.6 (0.439) |
|  | ♀ 4 x ♂ 19 | 1 | 1 |  | 12 | 12 | 24 | 0.5 |  | 0.75 | 6 | 18 | 8 (0.005) |
|  | ♀ 2 x ♂ 4 | 1 | 1 |  | 1 | 20 | 21 | 0.952 |  | 0.75 | 5.25 | 15.75 | 4.587 (0.032) |
|  | ♀ 5 x ♂ 7 | 0 | 1 |  | 3 | 14 | 17 | 0.824 |  | 0.5 | 8.5 | 8.5 | 7.118 (0.008) |
