# Supplementary material for: Population Genetic Structure of the Grasshopper Eyprepocnemis plorans in the South and East of the Iberian Peninsula
Source: PLoS One. 2013 Mar 8;8(3):e59041. doi: 10.1371/journal.pone.0059041 (PMC3592831; doi:10.1371/journal.pone.0059041)
Supplement: Table S8 — Population genetic parameters found with Hickory ( f = 0 model) with 87 loci, i.e. after removing the 10 ISSR markers showing the highest dropout values. Note that hs values are estimates of genetic diversity (panmictic or expected heterozygosity) for each population sample, whereas Hs is the average of hs values for all population samples. Ht is the heterozygosity that would be observed if all population samples would come from a single population. (DOC) [file pone.0059041.s012.doc]

| **Table S8 Population genetic parameters found with Hickory (*f*=0 model) with 87 loci, i.e. after removing the 10 ISSR markers showing the highest dropout values. Note that hs values are estimates of genetic diversity (panmictic or expected heterozygosity) for each population sample, whereas Hs is the average of hs values for all population samples. Ht is the heterozygosity that would be observed if all population samples would come from a single population.** | | | | |
| --- | --- | --- | --- | --- |
| **Parameter** | **Mean** | **SD** | **2.50%** | **97.50%** |
| theta-I, θ^(I)^ | 0.309 | 0.023 | 0.267 | 0.36 |
| theta-II, θ^(II)^ | 0.13 | 0.008 | 0.114 | 0.147 |
| theta-III, θ^(III)^ | 0.097 | 0.004 | 0.09 | 0.105 |
| hs[Algarrobo] | 0.223 | 0.005 | 0.213 | 0.234 |
| hs[Torrox] | 0.228 | 0.006 | 0.216 | 0.239 |
| hs[Nerja-0] | 0.219 | 0.005 | 0.208 | 0.229 |
| hs[Nerja-2] | 0.225 | 0.005 | 0.215 | 0.236 |
| hs[Salobreña] | 0.226 | 0.006 | 0.215 | 0.237 |
| hs[Mundo] | 0.232 | 0.007 | 0.217 | 0.246 |
| hs[Claras] | 0.212 | 0.007 | 0.199 | 0.225 |
| hs[Socovos] | 0.203 | 0.006 | 0.191 | 0.214 |
| hs[Calasparra] | 0.202 | 0.005 | 0.191 | 0.212 |
| hs[Caravaca] | 0.207 | 0.007 | 0.193 | 0.222 |
| Hs | 0.218 | 0.002 | 0.213 | 0.222 |
| Ht | 0.248 | 0.002 | 0.244 | 0.253 |
| Gst-B | 0.123 | 0.005 | 0.113 | 0.134 |

2.5% and 97.5% show confidence interval
