# Supplementary material for: Population Genetic Structure of the Grasshopper Eyprepocnemis plorans in the South and East of the Iberian Peninsula
Source: PLoS One. 2013 Mar 8;8(3):e59041. doi: 10.1371/journal.pone.0059041 (PMC3592831; doi:10.1371/journal.pone.0059041)
Supplement: Table S9 — Difference (%) in the population-genetics parameters estimated with 97 or 46 ISSR markers. Note that hs values are estimates of genetic diversity (panmictic or expected heterozygosity) for each population sample, whereas Hs is the average of hs values for all population samples. Ht is the heterozygosity that would be observed if all population samples would come from a single population. (DOC) [file pone.0059041.s013.doc]

| **Table S9 Difference (%) in the population-genetics parameters estimated with 97 or 46 ISSR markers**. **Note that hs values are estimates of genetic diversity (panmictic or expected heterozygosity) for each population sample, whereas Hs is the average of hs values for all population samples. Ht is the heterozygosity that would be observed if all population samples would come from a single population.** | | | |
| --- | --- | --- | --- |
| **Parameter** | **97 markers (a)** | **46 markers (b)** | **(a-b)/a** (%) |
| theta-I, θ^(I)^ | 0.299 | 0.369 | -23.41 |
| theta-II, θ^(II)^ | 0.129 | 0.159 | -23.26 |
| theta-III, θ^(III)^ | 0.098 | 0.106 | -8.16 |
| hs[Algarrobo] | 0.229 | 0.161 | 29.69 |
| hs[Torrox] | 0.23 | 0.178 | 22.61 |
| hs[Nerja-0] | 0.218 | 0.161 | 26.15 |
| hs[Nerja-2] | 0.228 | 0.181 | 20.61 |
| hs[Salobreña] | 0.229 | 0.172 | 24.89 |
| hs[Mundo] | 0.235 | 0.177 | 24.68 |
| hs[Claras] | 0.22 | 0.164 | 25.45 |
| hs[Socovos] | 0.205 | 0.167 | 18.54 |
| hs[Calasparra] | 0.21 | 0.176 | 16.19 |
| hs[Caravaca] | 0.216 | 0.185 | 14.35 |
| Hs | 0.222 | 0.172 | 22.52 |
| Ht | 0.253 | 0.204 | 19.37 |
| Gst-B | 0.123 | 0.156 | -26.83 |
