# Supplementary material for: Population Genetic Structure of the Grasshopper Eyprepocnemis plorans in the South and East of the Iberian Peninsula
Source: PLoS One. 2013 Mar 8;8(3):e59041. doi: 10.1371/journal.pone.0059041 (PMC3592831; doi:10.1371/journal.pone.0059041)
Supplement: Table S10 — Proportion of individuals from each population included into each group by Structure for 87 loci and K = 2. (DOC) [file pone.0059041.s014.doc]

| **Table S10 Proportion of individuals from each population included into each group by Structure for 87 loci and K=2** | | | |
| --- | --- | --- | --- |
| Population | No. individuals | Group 1 | Group 2 |
| Algarrobo | 29 | **0.938** | 0.062 |
| Torrox | 27 | **0.959** | 0.041 |
| Nerja-0 | 30 | **0.944** | 0.056 |
| Nerja-2 | 30 | **0.946** | 0.054 |
| Salobreña | 23 | **0.929** | 0.071 |
| Mundo | 15 | 0.197 | **0.803** |
| Claras | 21 | 0.065 | **0.935** |
| Socovos | 27 | 0.041 | **0.959** |
| Calasparra | 30 | 0.106 | **0.894** |
| Caravaca | 23 | 0.080 | **0.920** |

The highest assignment proportion to each group is marked in bold
