# Supplementary material for: Population Genetic Structure of the Grasshopper Eyprepocnemis plorans in the South and East of the Iberian Peninsula
Source: PLoS One. 2013 Mar 8;8(3):e59041. doi: 10.1371/journal.pone.0059041 (PMC3592831; doi:10.1371/journal.pone.0059041)
Supplement: Table S11 — Proportion of individuals from each population included into each group by Structure for 46 loci with K = 2. (DOC) [file pone.0059041.s015.doc]

| **Table S11 Proportion of individuals from each population included into each group by Structure for 46 loci with K=2** | | | |
| --- | --- | --- | --- |
| Population | No. individuals | Group 1 | Group 2 |
| Algarrobo | 29 | **0.970** | 0.030 |
| Torrox | 27 | **0.886** | 0.114 |
| Nerja-0 | 30 | **0.889** | 0.111 |
| Nerja-2 | 30 | **0.878** | 0.122 |
| Salobreña | 23 | **0.942** | 0.058 |
| Mundo | 15 | 0.084 | **0.916** |
| Claras | 21 | 0.064 | **0.936** |
| Socovos | 27 | 0.063 | **0.937** |
| Calasparra | 30 | 0.157 | **0.843** |
| Caravaca | 23 | 0.119 | **0.881** |

The highest assignment proportion to each group is marked in bold
