# Supplementary material for: Population Genetic Structure of the Grasshopper Eyprepocnemis plorans in the South and East of the Iberian Peninsula
Source: PLoS One. 2013 Mar 8;8(3):e59041. doi: 10.1371/journal.pone.0059041 (PMC3592831; doi:10.1371/journal.pone.0059041)
Supplement: Table S12 — Primers employed and number of markers obtained. (DOC) [file pone.0059041.s016.doc]

| **Table S12 Primers employed and number of markers obtained** | | |
| --- | --- | --- |
| Primer | Oligonucleotide Sequence (5´->3´) | No. markers |
| ISSR-6 | (CT)8-RG | 16 |
| ISSR-7 | (CTC)4-RC | 19 |
| ISSR-14 | (CT)8-RA | 11 |
| ISSR-26 | (CT)8-AC | 22 |
| ISSR-39 | (GA)8-YG | 18 |
| ISSR-43 | HVH-(TCC)5 | 11 |
| Total |  | 97 |
