# Supplementary material for: Population Genetic Structure of the Grasshopper Eyprepocnemis plorans in the South and East of the Iberian Peninsula
Source: PLoS One. 2013 Mar 8;8(3):e59041. doi: 10.1371/journal.pone.0059041 (PMC3592831; doi:10.1371/journal.pone.0059041)
Supplement: Table S13 — Summary of crosses and primers tested. (DOC) [file pone.0059041.s017.doc]

| **Table S13 Summary of crosses and primers tested** | | | | | |
| --- | --- | --- | --- | --- | --- |
|  |  |  | Primer | | |
| Cross | Population | Year | ISSR6 | ISSR7 | ISSR14 |
| ♀19 x ♂27 | Algarrobo | 2004 | x | x | x |
| ♀24 x ♂32 | “ | “ |  | x |  |
| ♀4 x ♂19 | “ | 2005 | x | x | x |
| ♀12 x ♂3 | “ | “ |  | x | x |
| ♀13 x ♂20 | “ | “ |  |  | x |
| ♀16 x ♂14 | “ | “ |  |  | x |
| ♀29 x ♂3 | “ | “ |  | x |  |
| ♀5 x ♂7 | Nerja-0 | 2004 |  | x | x |
| ♀2 x ♂4 | “ | 2005 |  |  | x |
| ♀3 x ♂3 | “ | “ |  | x |  |
