# Supplementary material for: Population Genetic Structure of the Grasshopper Eyprepocnemis plorans in the South and East of the Iberian Peninsula
Source: PLoS One. 2013 Mar 8;8(3):e59041. doi: 10.1371/journal.pone.0059041 (PMC3592831; doi:10.1371/journal.pone.0059041)
Supplement: Table S14 — Expected proportion of marker-carrying progeny for autosomal markers. 1 = marker presence; 0 = marker absence. (DOC) [file pone.0059041.s018.doc]

| **Table S14 Expected proportion of marker-carrying progeny for autosomal markers. 1= marker presence; 0= marker absence** | | | | | | | | |
| --- | --- | --- | --- | --- | --- | --- | --- | --- |
| Parent genotype | |  | Parent phenotype | |  |  |  |  |
| ♀ | ♂ |  | ♀ | ♂ |  | Expected progeny with phenotype 1 (%) | | |
| AA | AA |  | 1 | 1 |  | 100 | | |
| Aa | AA |  | 1 | 1 |  | 100 | | |
| aa | AA |  | 1 | 0 |  | 100 | | |
| AA | Aa |  | 1 | 1 |  | 100 | | |
| Aa | Aa |  | 1 | 1 |  | 75 | | |
| aa | Aa |  | 1 | 0 |  | 50 | | |
| AA | aa |  | 0 | 1 |  | 100 | | |
| Aa | aa |  | 0 | 1 |  | 50 | | |
| aa | aa |  | 0 | 0 |  | 0 | | |
