# Supplementary material for: Population Genetic Structure of the Grasshopper Eyprepocnemis plorans in the South and East of the Iberian Peninsula
Source: PLoS One. 2013 Mar 8;8(3):e59041. doi: 10.1371/journal.pone.0059041 (PMC3592831; doi:10.1371/journal.pone.0059041)
Supplement: Table S15 — Expected proportion of marker-carrying progeny for sex-linked markers. 1 = marker presence; 0 = marker absence. (DOC) [file pone.0059041.s019.doc]

| **Table S15 Expected proportion of marker-carrying progeny for sex-linked markers. 1= marker presence; 0= marker absence** | | | | | | | |
| --- | --- | --- | --- | --- | --- | --- | --- |
| Parent genotype | |  | Parent phenotype | |  | Expected progeny with phenotype 1 (%) | |
| ♀ | ♂ |  | ♀ | ♂ |  | ♀ | ♂ |
| X^+^ X^+^ | X^+^ |  | 1 | 1 |  | 100 | 100 |
| X^+^ X^+^ | X |  | 1 | 0 |  | 100 | 100 |
| X^+^ X | X^+^ |  | 1 | 1 |  | 100 | 50 |
| X^+^ X | X |  | 1 | 0 |  | 50 | 50 |
| X X | X^+^ |  | 0 | 1 |  | 100 | 0 |
| X X | X |  | 0 | 0 |  | 0 | 0 |
